# Impact of PREVENT Cardiovascular Risk Equations on Statin Eligibility by Subgroup and Risk Thresholds: A Cross-Sectional Study

**DOI:** 10.1007/s11606-025-09858-z

**Published:** 2025-09-25

**Authors:** Aileen P. Wright, Allison B. McCoy, Kimberly Garcia, Peter J. Embí, Walter K. Clair, MacRae F. Linton, Adam T. Wright

**Affiliations:** 1 Department of Biomedical Informatics, Vanderbilt University Medical Center, Nashville, TN USA; 2 Division of General Internal Medicine, Vanderbilt University Medical Center, Nashville, TN USA; 3 Division of Cardiovascular Medicine, Vanderbilt University Medical Center, Nashville, TN USA; 4 Department of Pharmacology, Vanderbilt University Medical Center, Nashville, TN USA

**Keywords:** atherosclerotic cardiovascular disease, prevention, risk prediction models, social deprivation index, statins

## Abstract

**Background:**

Replacing the pooled cohort equations (PCEs) with the Predicting Risk of Cardiovascular Disease EVENTs (PREVENT) equations for atherosclerotic cardiovascular disease (ASCVD) risk is projected to reduce statin eligibility, prompting discussion of lowering the risk threshold used with PREVENT. The potential impact on statin eligibility in different subgroups is unknown.

**Objective:**

To assess the impact of replacing PCEs with PREVENT equations on statin eligibility for a real-world population of primary care patients, incorporating social deprivation index (SDI) and lowering the ASCVD risk thresholds for statin eligibility.

**Design:**

Cross-sectional analysis comparing 10-year ASCVD risk scores and statin eligibility using the PCEs and PREVENT equations within a primary care population. Subgroup analyses were conducted by age, sex, and race. Risk thresholds for statin eligibility were varied to assess the effect on eligibility.

**Participants:**

Adult patients who visited a Vanderbilt primary care clinic in 2023.

**Main Measures:**

Estimated 10-year ASCVD risk and proportion of patients eligible for statin therapy using the PCEs vs. PREVENT equations.

**Key Results:**

In 50,123 patients, the mean 10-year ASCVD risk was significantly lower with PREVENT compared to the PCEs (3.6 vs. 7.5, *p* < 0.0001). In 36,430 patients not on statins, PREVENT reduced statin eligibility by 78.2%, with the largest reductions in women (82.6%), patients aged 40–49 (97.8%), and Black patients (81.2%). Lowering the statin eligibility threshold from 7.5 to 3% led to a 27.5% overall increase in eligibility rather than 78.2% reduction. However, gaps between subgroups expanded, and younger and Black patients retained relative reductions in eligibility (e.g., 4.7% decrease in statin eligibility among Black patients compared to a 32.7% increase among White patients).

**Conclusions:**

In a real-world primary care population, replacing the PCEs with the PREVENT equations would significantly reduce statin eligibility at the 7.5% threshold. Lowering the risk threshold would increase overall eligibility but disproportionately affect eligibility within certain subgroups.

**Supplementary Information:**

The online version contains supplementary material available at 10.1007/s11606-025-09858-z.

## INTRODUCTION

Cardiovascular disease remains the leading cause of death worldwide.^1^ Statins are an effective and underutilized therapy for preventing atherosclerotic cardiovascular disease (ASCVD) and reducing the incidence of cardiac events.^2,3^ For patients without an existing indication for statin therapy, clinical guidelines recommend calculating the 10-year ASCVD risk score to identify those most likely to benefit from risk reduction with statin therapy.^4^ The 2013 American College of Cardiology (ACC)/American Heart Association (AHA) pooled cohort equations.^5^ (PCEs) have been widely adopted by primary care physicians (PCPs) for this purpose. Clinical calculators incorporating the PCEs are accessed as web apps or electronic health record (EHR)–integrated risk calculators,^6^ or built into clinical decision support (CDS) tools, such as electronic reminders for statin prescribing in eligible patients.^7^

In 2023, the AHA developed the Predicting Risk of Cardiovascular Disease EVENTs (PREVENT) equations as an alternative for calculating 10-year ASCVD risk; equations were also developed for 30-year ASCVD risk, as well as heart failure and total cardiovascular risk.^8^ In recognition of concerns about the use of race in clinical algorithms,^9^ these equations removed race—a variable in the PCEs—and added a zip code-based social deprivation index (SDI) to capture social determinants of health. Additional variables, including kidney function, statin use, and hemoglobin A1c (HbA1c) level, were also added to enhance cardiometabolic risk prediction.

Studies using National Health and Nutrition Examination Surveys (NHANES) evaluating the potential impact of replacing the PCEs with the PREVENT equations have reported a significant decrease in both 10-year ASCVD risk scores and the percentage of patients eligible for statin therapy,^10–14^ resulting in an estimated 107,000 additional myocardial infarctions over 10 years if PREVENT were to replace the PCEs for statin and antihypertensive eligibility using the current 7.5% risk threshold.^11^ These analyses predicted the largest reductions in statin eligibility for older, male, and Black individuals, though they used publicly available data, which did not incorporate SDI. PREVENT has not yet been integrated into clinical guidelines for statin initiation. The lead developer of PREVENT and others have suggested lowering the threshold for statin eligibility when using PREVENT to, e.g., the 3 to 5% range, a controversial strategy which would help mitigate the large reduction in statin eligibility.^15,16^ The potential impact on population subgroups of lowering the threshold has yet to be investigated. We assessed the impact of applying the PCEs vs. PREVENT equations, incorporating SDI, to calculate 10-year ASCVD risk scores for a primary care population using EHR data. We also assessed statin eligibility and examined how changing the risk threshold from 7.5% to a lower value with the PREVENT equations would affect eligibility, overall and within subgroups defined by age, sex, and race.

## METHODS

### Study Population

This study was conducted at Vanderbilt University Medical Center (VUMC), a large urban academic medical center in Nashville, Tennessee. VUMC uses the Epic EHR which houses clinical data in a data warehouse, Clarity. We queried Clarity to extract clinical data available at the time a patient had a primary care visit, including patient demographics, laboratory data, medications, diagnoses, and variables required for the PCEs and PREVENT equations. Race information is collected from the patient at the time of registration, and can also be set by patients using the patient portal. This study was approved by the VUMC institutional review board.

**Figure 1:**
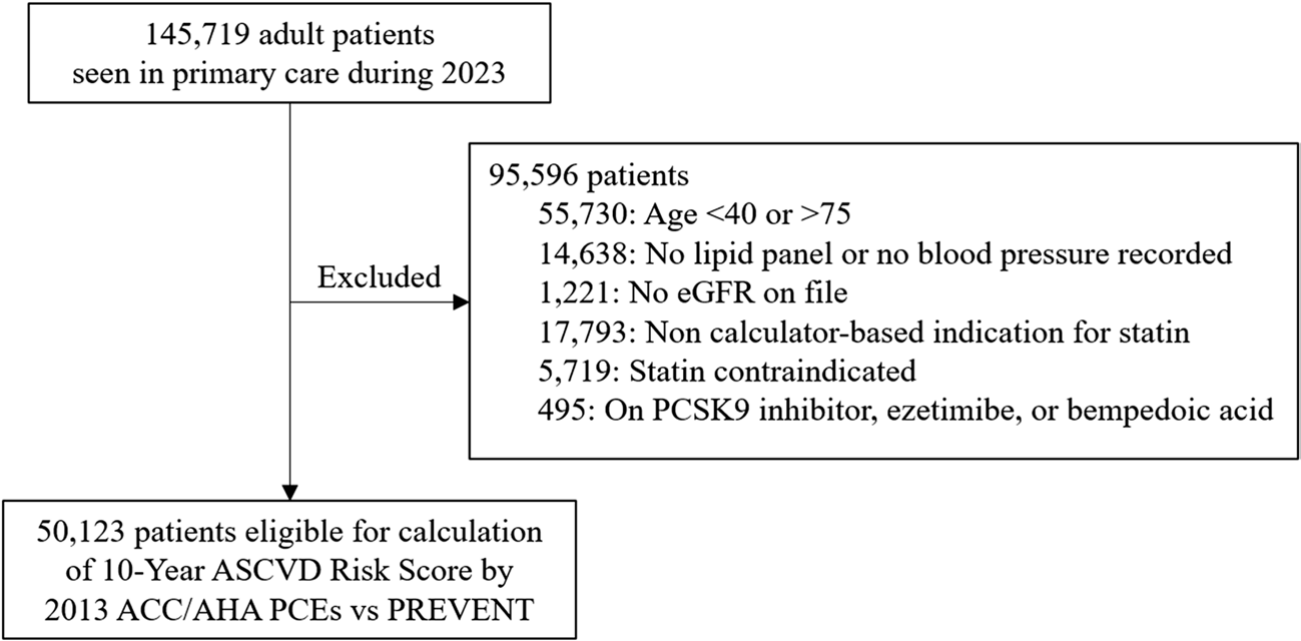
Study design. Note: eGFR, estimated glomerular filtration rate; PCSK9, Proprotein Convertase Subtilisin/Kexin Type 9; ASCVD, atherosclerotic cardiovascular disease; ACC, American College of Cardiology; AHA, American Heart Association; PCEs, pooled cohort equations; PREVENT, Predicting Risk of cardiovascular disease EVENTs.

Our study population consisted of all adult primary care patients seen in 2023 who were eligible for a calculator-based risk assessment to determine statin eligibility (Fig. 1). We excluded patients outside the 40 to 75 age range for the PCEs, and those without adequate data for the equations, such as patients without a lipid panel or blood pressure measurement on file. We excluded cases where patients had an existing indication for a statin regardless of 10-year ASCVD risk score, such as patients with a diagnosis of familial hypercholesterolemia or a low-density lipoprotein cholesterol (LDL-C) ≥ 190 mg/dL, an ASCVD diagnosis, or patients with diabetes over age 40. We also excluded patients for whom a statin was contraindicated due to pregnancy, lactation, a history of statin allergy or adverse effect of a statin, rhabdomyolysis, liver disease (decompensated liver disease, aspartate aminotransferase or alanine aminotransferase > 5 times upper limit of normal, or total bilirubin > 1.5 mg/dL), kidney disease (dialysis or estimated glomerular filtration rate (eGFR) < 10 ml/min/1.73 m^2^), or patients being provided comfort care at the end of life. Finally, we excluded patients on a PCSK9 inhibitor, bempedoic acid, or ezetimibe.

### Comparison of Atherosclerotic Cardiovascular Disease Risk Scores

For each included patient, we calculated the 10-Year ASCVD risk score using the PCEs and PREVENT equations. For patients on statins, we used an estimated pre-treatment total cholesterol level which we calculated using published average reductions in total cholesterol by statin formulation and dose, which we added to patients’ total cholesterol levels measured when on statin therapy, similar to other published methods (Supplemental Material).^3,10,17,18^ We then used this estimated pre-treatment total cholesterol level in both the PCEs and PREVENT equations, with the PREVENT statin variable set to 0. While the PCEs and PREVENT equations excluded patients with outlier values for continuous measurements, we included these patients but transformed their measurements to the calculator cutoffs, similar to other studies.^10^ The PREVENT equations include additional models for the optional variables of HbA1c level, urinary albumin-to-creatinine ratio (UACR), and zip code-based SDI. We used the full PREVENT model when at least 2 of these variables were available. If HbA1c was not available, we used the UACR model. If only SDI was available, we used the SDI model. If SDI was not available, we used the base model.

### Statistical Analysis

Descriptive statistics were used to summarize the demographic and clinical characteristics of the study population. Continuous variables were presented as mean values, while categorical variables were expressed as frequencies and percentages. We compared the mean 10-year ASCVD risk scores calculated with the PCEs vs. the PREVENT equations. Subgroup analyses were performed based on age, sex, and race to evaluate differences in risk scores and statin recommendations. To assess the reclassification of patients into different risk categories, we created contingency tables categorizing patients into low/borderline risk (< 7.5%), intermediate risk (7.5–19.9%), and high risk (> 20%) using both the PCEs and PREVENT. The percentage of patients reclassified into different risk categories was then calculated.

We also analyzed the impact of lowering the current 7.5% ASCVD risk score threshold on the percentage of patients for whom statin therapy would be recommended, corresponding to recent calls to lower the risk threshold used with PREVENT to, e.g., 3 to 5%.^15,16,19^ We evaluated thresholds from 7.5 to 3%. We then assessed the impact on different demographic subgroups, by determining the relative reduction or increase in statin recommendations for each group.

The statistical significance of differences in 10-year ASCVD risk scores between the PCEs and PREVENT was evaluated using paired *t*-tests. For subgroup analyses involving more than two groups, ANOVA was used, whereas *t*-tests were employed for comparisons between two groups. Pearson’s chi-square test was utilized to assess statistical significance within the contingency tables. A *p*-value of < 0.05 was considered statistically significant. All statistical analyses were conducted in R version 4.1.1.

## RESULTS

A total of 145,719 patients were seen in adult primary care at VUMC during 2023 (Fig. 1). After excluding patients for whom a 10-year ASCVD risk score would not be calculated to determine statin eligibility, such as patients with existing indications for or contraindications to a statin, or missing necessary data, our final cohort included 50,123 patients. Demographics for this population are shown in Table 1. Most patients were female (31,229, 62.3%) and White (40,812, 81.4%). 27.3% of patients were on a statin. The full PREVENT equations (which incorporate SDI) were used for 64.9% of patients and the SDI model for 34.7%, with the remaining using a less expanded PREVENT equation. Overall, 99.6% of patients had zip code-based SDI incorporated into their calculated PREVENT risk score.

**Table 1:** Characteristics of Included Patients and Differences in 10-Year ASCVD Risk Score

Demographics	No. (%)	PCE, mean	PREVENT, mean	Absolute score reduction with PREVENT
Age, years				
40–49	14,122 (28.2%)	2.1	1.2	0.9
50–59	14,692 (29.3%)	4.8	2.5	2.2
60–69	14,524 (29.0%)	10.3	4.9	5.4
70–75	6785 (13.5%)	18.8	8.1	10.7
Sex				
Female	31,229 (62.3%)	5.5	3.0	2.5
Male	18,894 (37.7%)	10.9	4.7	6.2
Race				
Black	5509 (11.0%)	8.5	3.8	4.6
Other	3802 (7.6%)	6.3	3.1	3.1
White	40,812 (81.4%)	7.5	3.6	3.9
Medications and conditions				
On statin	13,693 (27.3%)	12.6	5.8	6.8
Not on statin	36,430 (72.7%)	5.6	2.8	2.8
Smokers	3861 (7.7%)	12.5	5.1	7.5
On antihypertensive	24,611 (49.1%)	10.3	4.8	5.5
eGFR, mL/min/1.73 m^2^ < 60	3489 (7.0%)	11.6	6.5	5.1
Measurements, mean				
Systolic blood pressure	125.3			
eGFR, mL/min/1.73 m^2^	69.7			
Total cholesterol, mg/dL	192.9			
HDL cholesterol, mg/dL	56.3			
LDL cholesterol, mg/dL	114.2			
Total	50,123	7.5	3.6	3.9

The difference in the mean 10-year ASCVD risk scores between the PCE and PREVENT equations was statistically significant (*p* < 0.0001) both overall and within each subgroup

*PCE* pooled cohort equations, *PREVENT* Predicting Risk of Cardiovascular Disease EVENTs, *eGFR* estimated glomerular filtration rate, *HDL* high-density lipoprotein, *LDL* low-density lipoprotein

The mean 10-year ASCVD risk score was significantly lower using PREVENT compared to the PCEs (3.6 vs. 7.5, *p* < 0.0001). This difference was statistically significant across all age, sex, and racial groups, with the largest differences seen for the 70–75 age group (10.7), male patients (6.2), and Black patients (4.6). Overall, 5635 (11.2%) had a 10-year ASCVD risk score ≥ 7.5% by PREVENT compared to 18,820 (37.5%) by the PCEs, corresponding to a 70.1% relative reduction in patients in the intermediate or high risk category (Supplemental Table 2).

In 36,430 patients not on statins, overall statin eligibility was reduced by 78.2% with PREVENT vs. PCEs (2079 vs. 9541, *p* < 0.0001, Table 2). Of 9541 patients not on a statin and classified as intermediate or high-risk by the PCEs, 7483 (78.4%) lost a statin recommendation by being moved down to a low or borderline risk group with PREVENT. Of 26,889 patients who were low or borderline-risk patients by the PCEs, 21 (0.1%) gained a statin recommendation with PREVENT; on review, these patients had risk factors captured by PREVENT, but not the PCEs, such as kidney disease. Of 1478 patients eligible for high intensity therapy by the PCEs, 1146 (77.5%) were downgraded to moderate intensity with PREVENT, and 326 (22.1%) were moved to low or borderline risk; 6 (0.4%) still qualified for high intensity statin therapy.

**Table 2:** Comparison of Statin Risk Categories Using PCEs vs. PREVENT for Patients Not on Statins

By PCEs	10-year ASCVD risk category, no. (%) of patients			
	By PREVENT equations			
	Low or borderline risk, < 7.5%	Intermediate risk, 7.5%−19.9%	High risk, > 20%	Total
Low or borderline risk, < 7.5%	26,868 (73.8%)	21 (0.1%)	0 (0.0%)	26,889 (73.8%)
Intermediate risk, 7.5–19.9%	7157 (19.6%)	903 (2.5%)	3 (0.0%)	8063 (22.1%)
High risk, > 20%	326 (0.9%)	1146 (3.1%)	6 (0.0%)	1478 (4.1%)
Total	34,351 (94.3%)	2070 (5.7%)	9 (0.0%)	36,430 (100.0%)

*ASCVD* atherosclerotic cardiovascular disease, *PCEs* pooled cohort equations, *PREVENT* Predicting Risk of Cardiovascular Disease EVENTs

**Figure 2:**
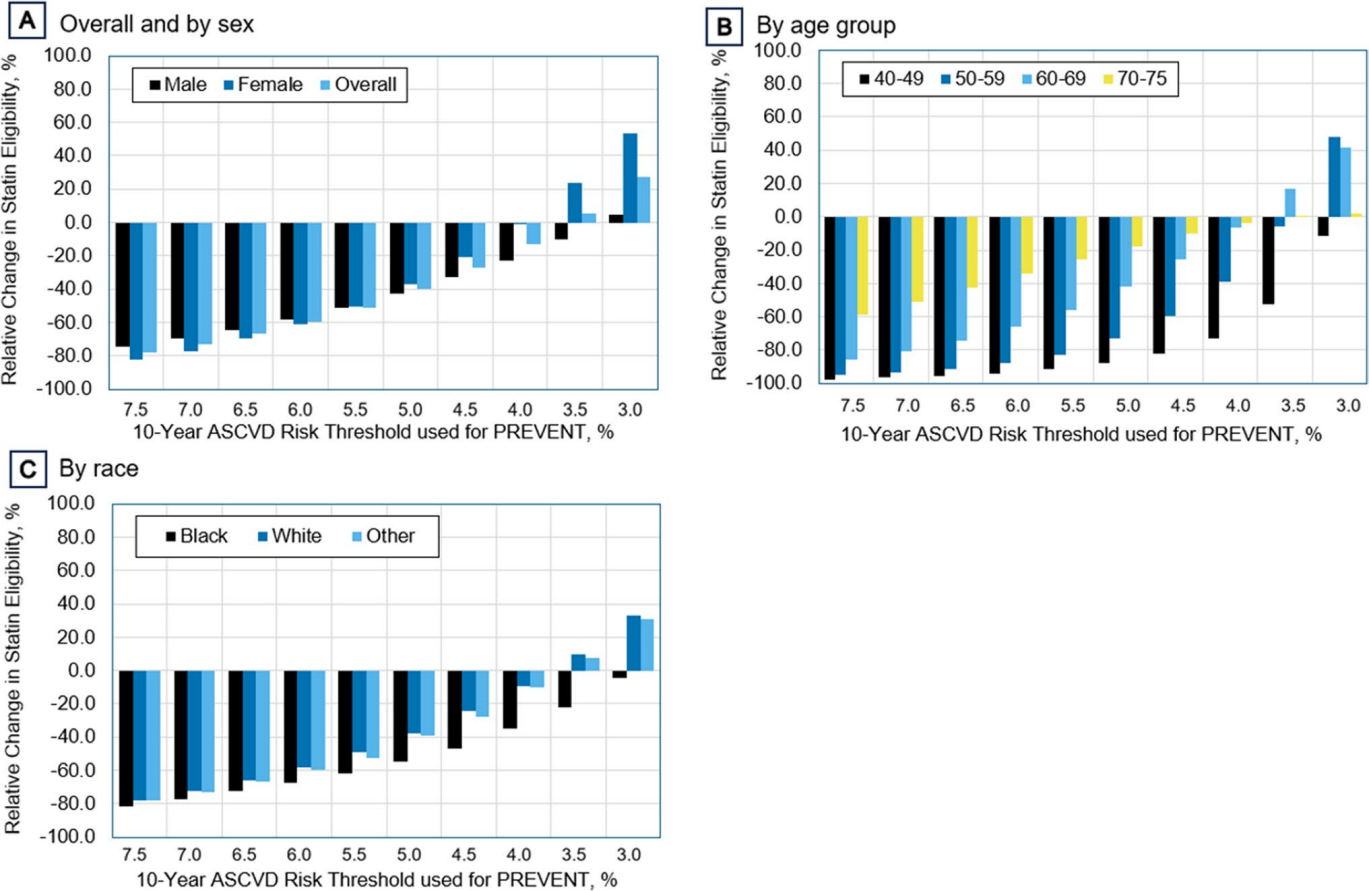
Change in statin eligibility for patients not on statins, across different ASCVD risk thresholds for PREVENT. Note: ASCVD, atherosclerotic cardiovascular disease; PCEs, pooled cohort equations; PREVENT, Predicting Risk of cardiovascular disease EVENTs.

When analyzed by subgroup, the greatest relative decreases in statin eligibility when using the current guideline-recommended 7.5% threshold were for women (82.6%), patients aged 40–49 (97.8%), and Black patients (81.2%) (Fig. 2). Moving the threshold for statin eligibility to 3% would lead to a 27.5% overall increase rather than 78.2% reduction in statin recommendations. The impact of using a 3% threshold varied for different subgroups, including a 5.0% increase in recommendations for men, a 53.7% increase for women, an 11.5% reduction for patients aged 40–49 and an increase of 48.1% for patients 50–59, and a 4.7% decrease for Black patients compared to a 32.7% increase for White patients. Overall, greater relative differences between subgroups occurred at the 3% threshold compared with the 7.5% threshold.

## DISCUSSION

In this study, we evaluated the impact of replacing the PCEs with the PREVENT equations in one primary care population using EHR data. Our finding that PREVENT significantly lowered 10-year ASCVD risk scores, especially in older, male, and Black patients, even when incorporating SDI, is consistent with prior studies using NHANES data.^10–14^ In our analysis of a cohort including patients both on and off statins, the mean 10-year ASCVD risk score was 3.6% with PREVENT vs. 7.5% with the PCEs, lower than previously published ranges (4.3–4.7% vs. 8.0–9.1%) This may be explained by population differences and our study’s use of PREVENT models incorporating zip code-based SDI, which lowered ASCVD risk scores in our population compared to the base PREVENT model. We found relative reductions in statin eligibility overall and for patients not on statins (70.1% and 78.2%, respectively), similar to previous reported ranges of 48.5 to 75.1%.^10–14^

Although the decrease in the mean 10-year ASCVD risk was greatest for older, male, and Black patients (Table 1 and Supplemental Table 1), relative reductions in statin recommendations were greatest for younger, female, and Black patients (Fig. 2 and Supplemental Fig. 1). This discrepancy likely reflects how close each subgroup’s 10-year risk scores by PCEs were to the 7.5% treatment threshold. For example, patients aged 70–75 had the greatest decrease in their mean 10-year ASCVD risk with PREVENT, but since they started out with high scores by PCEs (average = 18.8%), even large score reductions by PREVENT did not always bring them below 7.5%, resulting in a smaller relative reduction in statin recommendations in this subgroup compared to younger age groups. Strikingly, we observed greater between-group differences in eligibility reductions when the risk threshold was lowered to 3%, such as a 4.7% relative reduction in statin eligibility for Black patients compared to a 32.7% increase for White patients. This suggests that using PREVENT with a lower risk threshold could disproportionately impact subpopulations in a manner that is not readily apparent from analyses which use the 7.5% threshold.

Current discussion around whether to lower the risk threshold for statin eligibility used with PREVENT has revealed two major viewpoints.^15,16,19,20^ One perspective is that a reduction in statin prescriptions is a fitting change given the PCEs’ tendency to overestimate risk,^21^ infrequent but known side effects of statins such as muscle-related adverse events and diabetes, and the relatively high number needed to treat for statins, such as 78 for composite cardiovascular outcomes. ^20,22^ From this viewpoint, keeping the current risk threshold could refocus efforts to improve statin prescribing on a smaller population of high-risk individuals. Others argue that meta-analyses and randomized controlled trials demonstrate a net benefit of statin therapy for patients with risk as low as 3 to 5% over 10 years, and that use of a risk threshold as low as 3% would be cost-effective,^15,23^ leading some to conclude the statin risk threshold is very likely to be lowered when PREVENT is incorporated into newer guidelines.^24^ Given our findings that using PREVENT with a lower risk threshold may disproportionately reduce statin eligibility in certain subpopulations, further research should evaluate the impact of lowering the PREVENT risk threshold on eligibility among diverse populations.

Consensus that race is a social construct and its inclusion in algorithms may perpetuate systemic racism motivated the development of the race-free PREVENT equations for more equitable ASCVD prediction.^9^ In our analysis, similar to other studies,^10–14^ replacing PCEs with PREVENT lowered predicted risk and statin eligibility disproportionately among Black patients compared with other races. This finding raises the possibility that removing race from ASCVD risk prediction, while intended to promote equity, may paradoxically reduce prevention in the very populations it aims to protect—prompting the question of whether such changes are justified by the claim of improved overall accuracy. However, while the PCEs are known to overestimate risk and many studies have shown improved calibration with PREVENT, its “right-sizing” of risk may lead to underestimation in certain subpopulations. Indeed, one evaluation of PREVENT in the Multi-Ethnic Study of Atherosclerosis (MESA) found PREVENT reduced risk overestimation overall, but ultimately underestimated risk in White, Black, and Hispanic individuals.^25^ Given the fact that Black patients who are eligible for statins already have significantly lower rates of statin use than White patients,^26^ caution is warranted to avoid exacerbating disparities in ASCVD prevention in this population.

While race was removed from the PREVENT equations, zip code-based SDI was added to reflect geographic drivers of racial disparities.^9^ The incorporation of SDI into ASCVD risk prediction in the USA was a critical step which came after years of debate and advocacy around the incorporation of social risk in general and neighborhood deprivation in particular into cardiovascular disease risk prediction. Of note, other countries have already incorporated measures of socioeconomic status into CVD risk prediction, including the UK, which released QRISK, a CVD risk calculator including, among other variables, an area measure of deprivation, in 2007 and followed up with QRISK2 in 2008 which added self-identified ethnicity; both are included as risk factors in QRISK3. We found that incorporating zip code–based SDI lowered PREVENT 10-year ASCVD risk scores only in non-Black patients, reflecting that incorporation of SDI partially mitigates PREVENT’s disproportionate reduction in statin eligibility for Black patients. Nonetheless, relative decreases in ASCVD risk remained highest for Black patients even after incorporating SDI, and this gap expanded when the risk threshold for statin initiation was lowered. Of note, in the MESA population, the disparity in mortality between Black and White patients was reduced by adjusting for socioeconomic status but not eliminated.^27^ This raises a controversial question of whether the inclusion of race/ethnicity might improve the accuracy of ASCVD risk prediction. Relevant to reported mixed results on PREVENT’s calibration performance across different health systems,^28^ one study used machine learning to recalibrate PREVENT to a local population and demonstrated improved model calibration.^29^ While the authors refrained from including race in the recalibration of PREVENT, they reported improved calibration of the PCEs in Asian, Black, and Hispanic populations after including race. If the use of race as a variable improves model performance, it reflects the possibility that the social construct of race may function as an imperfect proxy for structural and environmental factors that contribute to clinical outcomes and are not captured by SDI.^30^

Our analysis was done in the context of incorporating a 10-year ASCVD risk calculator into an electronic reminder system for physicians when patients are eligible for statins. CDS tools like electronic reminders may increase guideline-adherent statin prescribing,^7^ and which risk calculator is incorporated into CDS tools has important implications for health systems.

Since publication of the 2013 ACC/AHA guidelines,^31^ the proportion of guideline-eligible adults who receive statins has plateaued at 35%.^3^ Undertreatment of at-risk individuals remains a major challenge in heart disease prevention,^32^ and increasing statin prescribing for eligible patients is a major quality improvement focus.^7^ Beyond statins, adoption of PREVENT for ASCVD risk determination would also have implications for blood pressure management,^33^ aspirin for primary prevention,^4^ and potentially decision-making around eligibility for other medications such as GLP-1 receptor agonists.^34^ Further work is needed to determine how to integrate updated ASCVD risk prediction models into clinical care without inadvertently reducing appropriate statin prescribing for prevention.

### Limitations

Our study assessed the impact of using the PREVENT equations on CDS recommendations at one institution with a Black population of 11%. More work needs to be done to assess the impact in different populations and settings. In addition, racial data recorded in the EHR are known to have inaccuracies; in practice, these data are still used to drive CDS tools.

## CONCLUSIONS

When applied to a real-world primary care population, replacing the PCEs with the PREVENT equations used with a 7.5% threshold significantly reduced statin eligibility. Lowering the risk threshold would increase overall eligibility but disproportionately affect eligibility within certain subpopulations. More work is needed to determine how to incorporate PREVENT into clinical care without negatively impacting subpopulations who might benefit from increased statin prescribing.

## Supplementary Information

Below is the link to the electronic supplementary material.

Supplementary file (DOCX 97.2 KB)

## Data Availability

The dataset generated and analyzed in this study contains patient information and cannot be shared publicly to protect participant privacy. Deidentified data may be made available from the corresponding author on reasonable request, in accordance with institutional policies.
