# Supplementary material for: Impact of PREVENT Cardiovascular Risk Equations on Statin Eligibility by Subgroup and Risk Thresholds: A Cross-Sectional Study
Source: J Gen Intern Med. 2025 Sep 25;41(8):2248–55. doi: 10.1007/s11606-025-09858-z (PMC13241340; doi:10.1007/s11606-025-09858-z)
Supplement: Supplementary file 1 — Supplementary file (DOCX 97.2 KB) [file 11606_2025_9858_MOESM1_ESM.docx]

Supplementary Materials

Methods

Estimation of Pretreatment ASCVD Risk Among Patients on Statins

For patients on statins, we calculated estimated pre-treatment total cholesterol levels. This was done by using published average reductions in total cholesterol by statin formulation and dose, and adding these to patients’ total cholesterol levels measured when on statin therapy, similar to other published methods^3,10,17,18^. We did not adjust HDL levels due to insignificant effects of statins on HDL levels in meta-analyses.

| **Statin** | **Dose (mg)** | **Total Cholesterol (mg/dL)** |
| --- | --- | --- |
| Atorvastatin | 6-10 | -49.49 |
|  | 11-20 | -66.75 |
|  | 21-40 | -71.55 |
|  | >40 | -79.22 |
| Fluvastatin | 11-20 | -19.82 |
|  | 21-40 | -26.27 |
|  | >40 | -28.41 |
| Lovastatin | ≤10 | -34.66 |
|  | 11-20 | -40.21 |
|  | 21-40 | -24.94 |
|  | >40 | -67.26 |
| Pravastatin | 6-10 | -30.07 |
|  | 11-20 | -38.61 |
|  | 21-40 | -41.16 |
|  | >40 | -27.28 |
| Rosuvastatin | ≤5 | -29.03 |
|  | 6-10 | -61.49 |
|  | 11-20 | -72.14 |
|  | >20 | -87.75 |
| Simvastatin | 6-10 | -49.48 |
|  | 11-20 | -56.93 |
|  | 21-40 | -60.26 |
|  | >40 | -81.94 |
| Pitavastatin | 1 | -67.6 |
|  | 2 | -67.2 |
|  | 4 | -83.6 |

Supplemental Figure 1. Change in statin eligibility across different ASCVD risk thresholds for PREVENT, including patients on and off statin therapy.


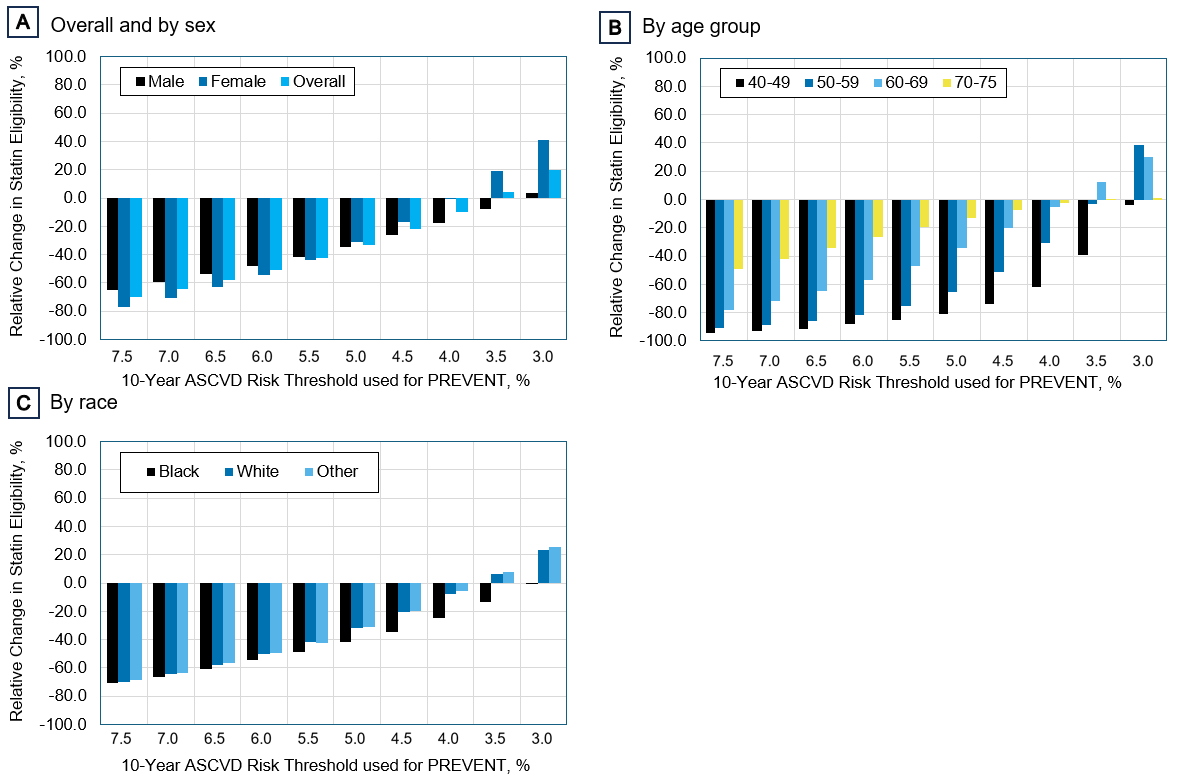


Supplemental Table 1. Characteristics of Included Patients and Differences in 10-Year ASCVD Risk Score for patients not on statins

| **Demographics** | **No. (%)** | **PCE, mean** | **PREVENT, mean** | **Absolute score reduction with PREVENT** |
| --- | --- | --- | --- | --- |
| Age, years |  |  |  |  |
| 40-49 | 12,715 (34.9% | 1.8 | 1.1 | 0.7 |
| 50-59 | 11,329 (31.1% | 4.0 | 2.2 | 1.8 |
| 60-69 | 9,026 (24.8% | 8.8 | 4.3 | 4.5 |
| 70-75 | 3,360 (9.2% | 17.3 | 7.3 | 10.0 |
| Sex |  |  |  |  |
| Female | 24,245 (66.6% | 4.2 | 2.4 | 1.8 |
| Male | 12,185 (33.4% | 8.4 | 3.6 | 4.8 |
| Race |  |  |  |  |
| Black | 3,963 (10.9% | 6.6 | 2.8 | 3.8 |
| Other | 2,878 (7.9% | 4.6 | 2.4 | 2.2 |
| White | 29,589 (81.2% | 5.6 | 2.8 | 2.8 |
| **Medications and conditions** |  |  |  |  |
| Smokers | 2,495 (6.8% | 9.9 | 4.0 | 5.9 |
| On antihypertensive | 15,038 (41.3% | 7.8 | 3.7 | 4.1 |
| eGFR, mL/min/1.73 m^2^ <60 | 2,022 (5.6% | 9.1 | 4.9 | 4.2 |
| **Measurements, mean** |  |  |  |  |
| Systolic blood pressure | 124.4 |  |  |  |
| eGFR, mL/min/1.73 m^2^ | 69.7 |  |  |  |
| Total cholesterol | 198.3 |  |  |  |
| HDL cholesterol, mg/dL | 57.7 |  |  |  |
| LDL cholesterol, mg/dL | 118.6 |  |  |  |
| **Total** | 36,430 | 5.6 | 2.8 | 2.8 |

Note: The difference in the mean 10-year ASCVD risk scores between the PCE and PREVENT equations was statistically significant (p < 0.0001) both overall and within each subgroup. Abbreviations: PCE, Pooled Cohort Equations; PREVENT, Predicting Risk of Cardiovascular Disease EVENTs; eGFR, estimated Glomerular Filtration Rate; HDL, High-Density Lipoprotein; LDL, Low-Density Lipoprotein.

Supplemental Table 2. Comparison of Statin Risk Categories Using PCEs vs. PREVENT for all patients

|  | **10-year ASCVD risk category, No. (%) of patients** | | | |
| --- | --- | --- | --- | --- |
|  | **By PREVENT equations** | |  |  |
| **By PCEs** | Low or borderline risk, <7.5% | Intermediate risk, 7.5%-19.9% | High risk, >20% | Total |
| Low or borderline risk, <7.5% | 31,241 (62.3%) | 62 (0.1%) | 0 (0.0%) | 31,303 (62.5%) |
| Intermediate risk, 7.5%-19.9% | 12,473 (24.9%) | 2,261 (4.5%) | 26 (0.1%) | 14,760 (29.4%) |
| High risk, >20% | 774 (1.5%) | 3,231 (6.4%) | 55 (0.1%) | 4,060 (8.1%) |
| Total | 44,488 (88.8%) | 5,554 (11.1%) | 81 (0.2%) | 50,123 (100.0%) |

Abbreviations: ASCVD, Atherosclerotic Cardiovascular Disease; PCEs, Pooled Cohort Equations; PREVENT, Predicting Risk of Cardiovascular Disease Events.

Supplemental Table 3. Comparison of Statin Risk Categories Using PCEs vs. PREVENT for Patients on Statins

|  | **10-year ASCVD risk category, No. (%) of patients** | | | |
| --- | --- | --- | --- | --- |
|  | **By PREVENT equations** | |  |  |
| **By PCEs** | Low or borderline risk, <7.5% | Intermediate risk, 7.5%-19.9% | High risk, >20% | Total |
| Low or borderline risk, <7.5% | 4,373 (31.9%) | 41 (0.3%) | 0 (0.0%) | 4,414 (32.2%) |
| Intermediate risk, 7.5%-19.9% | 5,316 (38.8%) | 1,358 (9.9%) | 23 (0.2%) | 6,697 (48.9%) |
| High risk, >20% | 448 (3.3%) | 2,085 (15.2%) | 49 (0.4%) | 2,582 (18.9%) |
| Total | 10,137 (74.0%) | 3,484 (25.4%) | 72 (0.5%) | 13,693 (100.0%) |

Abbreviations: ASCVD, Atherosclerotic Cardiovascular Disease; PCEs, Pooled Cohort Equations; PREVENT, Predicting Risk of Cardiovascular Disease EVENTs.
